# Respiratory monitoring and apnoea detection in paediatric and neonatal patients using a wearable accelerometer-based chest sensor: protocol for an observational diagnostic feasibility study

**DOI:** 10.1136/bmjopen-2025-104363

**Published:** 2025-08-31

**Authors:** Hannah Vennard, Elise Buchan, Jennifer Miller, Stuart Kelly, Catriona Cowan, Osian Meredith, Bruce Henderson, David J Lowe, Neil Patel, Sameer Zuberi, Ross Langley

**Affiliations:** 1 Department of Paediatric Respiratory and Sleep Medicine, Royal Hospital for Children, Glasgow, UK; 2 Medicine Veterinary and Life Science, University of Glasgow, Glasgow, UK; 3 PneumoWave Ltd, Glasgow, UK; 4 Neonatal Department, Royal Hospital for Children, Glasgow, UK; 5 Department of Neurology, Royal Hospital for Children, Glasgow, UK

**Keywords:** Epilepsy, SLEEP MEDICINE, Cot death, Observational Study, Respiratory physiology

## Abstract

**Abstract:**

**Introduction:**

Accurate evaluation of respiratory rate and pattern is important in health and disease; however, it can be challenging in children and babies due to small size and poor tolerability of existing monitoring equipment. This protocol outlines a study evaluating the feasibility of collecting respiratory data using a chest-worn accelerometer-based motion sensor in paediatric patients at risk of apnoea, respiratory failure and sudden death.

**Methods and analysis:**

This is an observational feasibility study over a 2-year period. The biosensor is an accelerometer worn on an ECG electrode during standard care at the Royal Hospital for Children, Glasgow. We aim to recruit three groups of patients (75 patients to each group): (1) Children attending for overnight cardiorespiratory polygraphy (0 to ≤16 years), (2) neonatal inpatients (from 30 weeks gestation) and (3) children attending for video telemetry at the epilepsy monitoring unit (0 to ≤16 years). Measurements will include (1) chest and/or abdominal wall motion measured by the worn biosensors; (2) standard clinical monitoring data will be collected to support biosensor data interpretation and (3) acceptability will be measured by a feedback questionnaire completed by patients and their parents/guardians.

**Ethics and dissemination:**

This study protocol was reviewed and approved by Yorkshire and The Humber-Leeds East Research Ethics Committee, approval number 314 696. This study involves human participants; written informed consent and assent, when appropriate, will be obtained from participants (or their parent/legal guardian/next of kin) to participate in the study. The study will be carried out in accordance with the World Medical Association Declaration of Helsinki (1964) standard reporting. Study findings will be reported clearly and transparently with relevant stakeholders including researchers, practitioners and publicly available databases. Results from this study will be presented at national and international conferences and reported in peer-reviewed publications.

**Trial registration number:**

NCT06292299 pre-results stage.

**STRENGTHS AND LIMITATIONS OF THIS STUDY**

- This study involves data collection from paediatric patients wearing a novel biosensor in a tertiary children’s hospital, with a broad eligibility criteria strengthening external validity.
- The biosensor data can be paired with standard clinical monitoring data to provide a comprehensive ground truth for data interpretation.
- Children recruited to this study are at risk of apnoea, sudden death and respiratory failure, a population that may benefit from wearable technology, which can identify changes in respiratory metrics.
- Movement artefact has the potential to impact the visualisation of the respiratory waveform; however, actigraphy as a metric can have value in monitoring the clinical condition.

## Introduction

 Novel wearable technology has the potential to improve the accessibility and tolerability of respiratory monitoring for paediatric and neonatal patients.^1^ This observational study explores the feasibility of combining a wireless wearable device and data science to support respiratory evaluation in different paediatric patient groups. Longitudinal data collection using wearable technology may provide novel insights into pathophysiology of conditions where abnormal respiratory function or mechanics are suspected of contributing to patient morbidity. Continuous respiratory monitoring can allow identification of signatures and trends warning of clinical deterioration or impending apnoea. The COVID-19 pandemic highlighted the value of wearable technology for remote monitoring and automated diagnosis harnessing advances in technology and artificial intelligence.^1^

Sleep disordered breathing (SDB) in children is common, with prevalence estimated to be around 2%–5% and can have implications for a child both in terms of physical health and neurocognitive development.^2^,^9^ Untreated, SDB is associated with increased risk of cardiovascular and metabolic disease, as well as developmental, learning and behavioural issues.^10^,^13^ Gold standard paediatric sleep diagnostics requires complex multichannel tests in specialised centres, limiting access and availability, resulting in delayed diagnosis and management.^14^ Early identification and treatment of SDB has been shown to improve outcomes.^15^ SDB is more prevalent among children with genetic syndromes and neurodevelopmental disorders, who can find gold standard diagnostic evaluation challenging.^16^,^19^ The nasal cannula and thermistor have been identified as most challenging to tolerate, particularly for younger children.^20^ The reliance on diagnostics equipment which is not acceptable to a patient population leads to health disparities.

Evaluating respiratory rate (RR) and pattern in neonatal patients is a crucial part of routine monitoring of the newborn. RR is also an indicator of respiratory distress and apnoea of prematurity which can have neurodevelopmental consequences if not identified and treated appropriately.^21 22^ Respiratory monitoring in neonatal patients can be challenging due to their small size, higher RR and low tidal volume (TV). There is consequently a need for lightweight wireless wearable technology suitable for neonatal patients.^23^,^25^ Similarly, newborn term babies, which are typically unmonitored on postnatal wards, can have an unexpected collapse which, although rare, can have catastrophic outcomes.^26 27^ Controversies exist around home monitoring for sudden unexpected death in infants; however, cardiorespiratory monitoring following a previous brief resolved unexplained event can be considered.^28^

Children with epilepsy are at increased risk of sudden unexpected death in epilepsy (SUDEP), the pathophysiology of which remains unclear; however, recent evidence suggests that terminal events are commonly preceded by central apnoea.^29^,^31^ Improving our understanding of SUDEP is fundamental for prevention and development of an alert system for family members or healthcare providers. The landmark Mortality in Epilepsy Monitoring Unit Study showed that resuscitative efforts were delayed in SUDEP in epilepsy monitoring units.^29^ Resuscitation was initiated within 3 min of cardiorespiratory arrest in non-fatal cases but delayed by at least 10 min in fatal cases. In epilepsy syndromes, such as Dravet’s, 20% of individuals die before 20 years old; 50% of these from SUDEP.^32^,^34^ An alarm and rapid response to children who have apnoea in association with seizures could save lives (1.2/1000 deaths per year in UK).^29 35^

**Figure 1:**
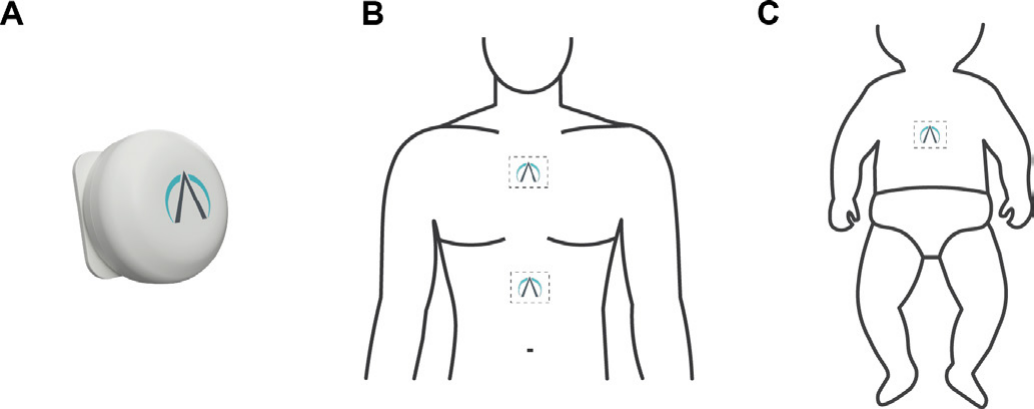
PneumoWave biosensor and placement on the body. (A) Image of biosensor. (**B**) Location of biosensor placement for attending for evaluation of sleep disordered breathing will wear a chest and abdominal biosensor to allow for evaluation of paradoxical thoracoabdominal excursions (paradoxical breathing). (C) Location of biosensor placement for neonatal patient. Patients with epilepsy attending for video telemetry will wear a chest biosensor.

The PneumoWave biosensor is a small device (40 mm in diameter and 14 mm in height, 20 g in weight) that attaches onto the chest/abdomen using an ECG electrode and is intended to capture and store chest motion data continuously over a period shown in figure 1. The device will be used in a research application to assess activity where quantifiable analysis of physical motion is desirable and has the potential to be used to detect changes in breathing patterns, as well as detect reduced chest movement and cessation of breathing. During this study, we aim to determine the feasibility of collecting respiratory waveform data from three groups of paediatric patients at risk of apnoea, where accurate monitoring of RR and pattern can be challenging.

This study aims to (1) determine the feasibility of collecting respiratory data from wearable accelerometer-based biosensor in children attending for cardiorespiratory polygraphy (CR-P) sleep studies, neonatal patients and children with epilepsy attending for video telemetry (VT), (2) interrogate biosensor data in line with standard clinical monitoring to assess potential for RR, pattern and apnoeas, (3) explore patients’ and parents’ acceptability of the wearable biosensor, (4) identify novel clinical pathway candidates which include accelerometry to address unmet needs in paediatric diagnostics and longitudinal clinical monitoring and (5) capture longitudinal data from children with conditions associated with unexpected collapse to identify digital biomarker candidates for future research.

## Methods and analysis

### Study design

This is an observational diagnostic feasibility study over a 2-year period aiming to collect biosensor data from 75 patients in each of the three groups: CR-P (sleep study), neonates and VT (epilepsy). Patients who consent to take part will be assigned a study number which will be linked to demographic information as well as standard clinical monitoring data.

### Eligibility criteria

#### Inclusion criteria

Patients in the CR-P group must be (1) undergoing CR-P, (2) ≤16 years old and (3) patients and/or parents/guardians must speak and read English. Patients in the epilepsy group patients must be (1) attending for VT, (2) ≤16 years old and (3) patients and/or parents/guardians must speak and read English. Patients in the neonatal group must be (1) staying in the neonatal unit or postnatal wards, (2) ≥30 weeks’ gestation and (3) parents/guardians who speak and read English.

#### Exclusion criteria

Exclusion criteria for all three groups include (1) outside age range, >16 years of age for CR-P/VT patients or <30 weeks corrected gestation for neonatal patients, (2) broken skin over chest/abdominal area, (3) sensitivity to ECG electrodes, (4) implanted pacemaker device in situ and (5) in the clinical opinion of the investigator, the patient is not suitable for inclusion.

### Objectives

The primary objective of the study is to assess the feasibility of collecting respiratory waveform data using the PneumoWave biosensor in children attending for CR-P, neonatal inpatients on a range of respiratory support, including those unsupported and patients with epilepsy attending for VT. The secondary objective is to view biosensor respiratory waveform alongside standard clinical monitoring data. We will collect data on biosensor acceptability from the patient (as able) and parent/guardian.

### Outcome measures

#### Primary outcome measures

Feasibility of collecting respiratory waveform data using PneumoWave biosensor in paediatric patients attending for CR-P, VT and neonatal patients. (1) We will quantify the length of time the device is on each patient compared with monitoring time, (2) if the device is removed by the patient/other and (3) the ability of the device to collect data while in situ.

#### Secondary outcome measures

Secondary outcomes include collating respiratory data from standard clinical monitoring and viewing biosensor data to evaluate potential for PneumoWave to evaluate normal and abnormal RR/pattern. Standard clinical monitoring data will be used to provide a ‘ground truth’ to support algorithm development and refinement. Examples of standard clinical data include RR count by clinical team, respiratory inductance plethysmography (RIP) band waveform data from CR-P, ventilator measured parameters including RR and TV, apnoeas scored by physiologists from CR-P. We will observe different types of respiratory events, including obstructive and central apnoea and hypopnoea, as well as respiratory pattern changes, including periodic breathing and hypoventilation reflected in elevated transcutaneous carbon dioxide (TcCO_2_).

#### Acceptability

We will assess the acceptability of the biosensor by providing feedback questionnaires to patients and parents at the end of the monitoring period. Feedback forms have been developed for children 6–11 years of age and 12–16 years of age.

### Data collection process and study timeline

**Figure 2:**
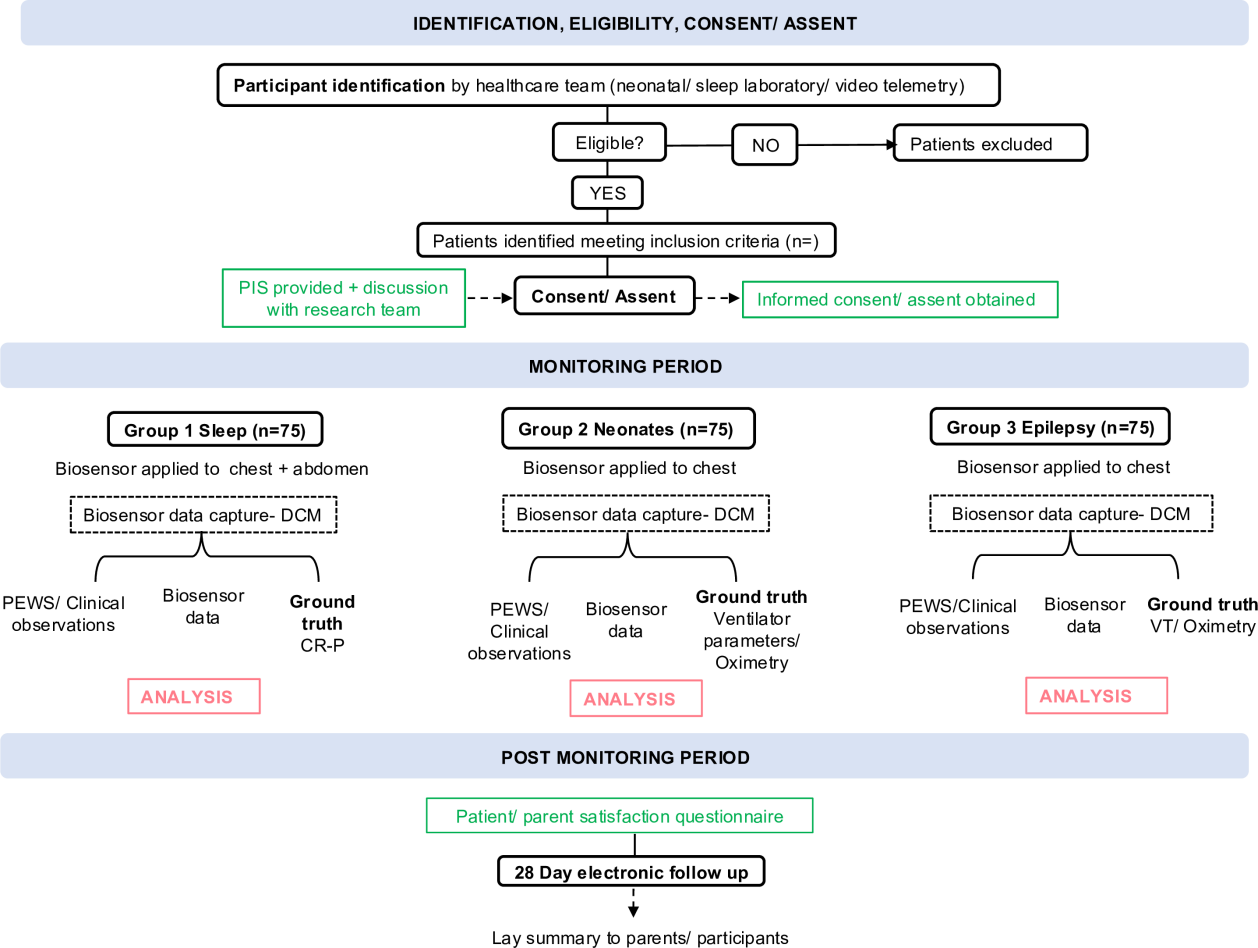
On-site workflow for observational study. Group 1 includes children attending for cardiorespiratory polygraphy; group 2 includes neonates staying in the neonatal unit or postnatal ward and group 3 includes children attending the epilepsy monitoring unit for video telemetry. Green boxes represent ethics forms. CR-P, cardiorespiratory polygraphy; DCM, data capture mobile; PEWS, Paediatric Early Warning Score; PIS, participant information sheet; VT, video telemetry.

Patients will be identified by the usual care team and consented for participation, as shown in figure 2 and table 1. Families expressing interest in the study will be approached by a member of the research team who answered any questions, and written informed consent/assent will be obtained when appropriate, as shown in figure 2 and table 1.

**Table 1:** Data sources

Patient group	PneumoWave sensors worn	Standard clinical monitoring data	Outcome of interest
Cardiorespiratory sleep study patients	Chest and abdominal sensor	Thoracic and abdominal RIP channels, RIP sum, Oximetry (SpO_2_ and HR), ECG, body position, movement and nasal pressure flow (all children), video, audio and TcCO_2_ (some children)	Tolerability of PneumoWave device Feasibility of data collection from PneumoWave device RR Apnoeas and hypopnoeas (obstructive, central, mixed)
Neonatal patients	Chest sensor	Oximetry (SpO_2_ and HR)	Tolerability of PneumoWave device Feasibility of data collection from PneumoWave device RR Central apnoeas/periodic breathing
Epilepsy patients attending for video telemetry	Chest sensor	Oximetry (SpO_2_ and HR) Video EEG Feedback forms completed by parents and children >5 years of age	Tolerability of PneumoWave device Feasibility of data collection from PneumoWave device RR Central apnoeas in peri-ictal period Respiratory pattern in peri-ictal period Parent/patient feedback on biosensor acceptability

EEG, electroencephalogram; HR, heart rate; RIP, respiratory inductance plethysmography; RR, respiratory rate; SpO_2_, peripheral oxygen saturation; TcCO_2_, transcutaneous carbon dioxide.

### Data collection from patient groups

**Figure 3:**
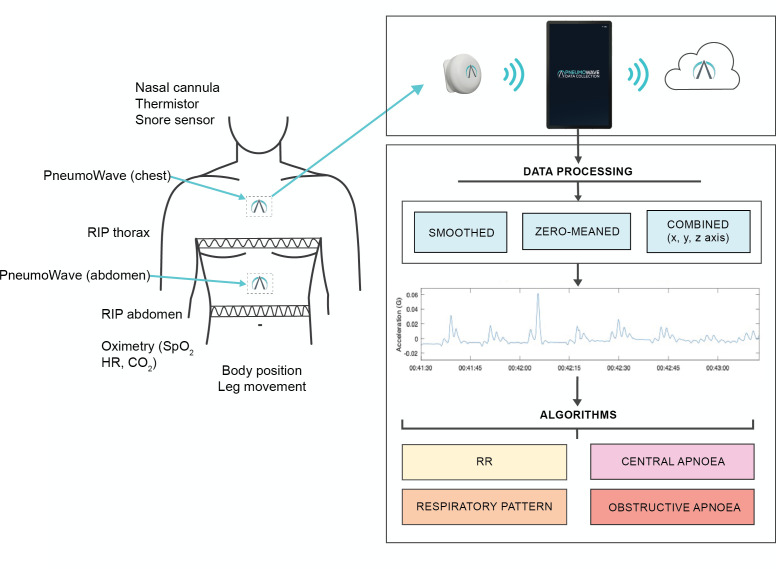
Data collection using novel biosensors in CR-P patients. Children attending for CR-P will wear a chest and abdominal biosensor attached using ECG electrodes. Biosensors are positioned in the midline next to RIP bands. CR-P equipment will be worn as per standard clinical practice. Accelerometer data are transferred via Bluetooth from each biosensor to an application on a mobile device. Data are then stored in a cloud. Data downloaded from the cloud (x, y, z axis) are processed, smoothed, zero meaned and combined to generate a respiratory waveform. CR-P, cardiorespiratory polygraphy; CO_2_, carbon dioxide; HR, heart rate; RIP, respiratory inductance plethysmography; RR, respiratory rate; SpO_2_, peripheral oxygen saturation.

Patients attending for CR-P will wear a chest and abdominal biosensor shown in figure 3 and tables1  2. CR-P is recorded using SOMNOscreen (SOMNOmedics, Germany). Respiratory effort is measured using thoracic and abdominal RIP bands, alongside the derived RIP sum channel for surrogate flow analysis if required. Oxygen saturation (SpO_2_) and pulse rate are measured by Nonin integrated probe. ECG, body position, movement and nasal pressure flow are routinely recorded, while video, audio and TcCO_2_ are recorded in some children. CR-P recordings will be scored by sleep physiologists according to American Academy of Sleep Medicine (AASM) guidelines used to diagnose SDB, allowing identification of different types of apnoeas and hypopneas (obstructive, central, mixed), breathing patterns including periodic breathing and hypoventilation.^36^

**Table 2:** Summary of study procedures

Study procedure	Screening/consent (first visit)	Research episode	28 days follow-up electronically
Obtain informed consent where relevant	√		
Review inclusion/exclusion criteria	√		
Complete case report form*	√	√	√
Collect continuous data from standard care vital sign monitor		√	
Collect waveform respiratory data		√	
Review retrospective notes for adverse outcome			√

* Demographics (gender, age, ethnicity), medical history, sleep history, medication history, date/time of monitoring period, position of sensor on body and position patient (supine, prone, right/left lateral) and concurrent activities (eg, feeding, nappy changing, being held by parents), adverse event monitoring, patient and parent satisfaction form.

The PneumoWave data capture mobile (DCM) system consists of a wearable biosensor and a data collecting app running on an Android mobile platform and a cloud storage and viewing dashboard. Accelerometer data can also be downloaded from the portal for analysis. Acceleration data will be transferred to a secure cloud for storage and data analysis.

Patients in the neonatal unit will wear a single chest sensor in addition to standard clinical monitoring for a period of 1–6 hours (table 1). Neonates in the postnatal wards are largely unmonitored and therefore a Masimo Radical 97 will be used to collect concurrent SpO_2_ (oxygen saturation), heart rate and RRp (respiratory rate derived from plethysmography waveform) data. Patients in the special care baby unit who may be receiving respiratory support (including oxygen therapy, high flow oxygen therapy or non-invasive positive airway pressure) will have continuous oximetry monitoring. Neonates in intensive care may be receiving invasive mechanical ventilation of different modes supporting patient-triggered breaths (patient-triggered ventilation, synchronised intermittent mandatory ventilation) to mandatory breaths. Ventilator measured parameters including RR, TV as well as inspiratory time as well as inspiratory time, peak inspiratory pressure and positive end expiratory pressure will be recorded.

Patient attending for VT assessment will wear a single chest biosensor in addition to electroencephalogram (EEG) (Natus, Neuroworks software) video and Masimo Radical-97 or Masimo Radical-7 touch oximetry monitoring for the duration of their stay (1–4 days) (table 1).

During the monitoring period, case report forms (CRFs) will be completed as shown in table 2. CRFs were designed to capture data on participant demographics, medical history, current medication, information related to biosensor monitoring including time/duration of monitoring, position of biosensor on patient, patient position (supine, prone, right or left lateral), events or patient activities during monitoring (eg, breastfeeding, held by parents), and whether monitoring was tolerated by the participant.

### Sample size

This is a feasibility study to generate data for future prospective interventional studies. A total of 225 patients will be recruited with an aim of 75 patients from each study group. 75 patients per group was decided to be an appropriate number to provide a range of apnoea types, range of comorbidities, demographics and to evaluate tolerability across a diverse patients group representative of patient population to support external validity.

### Statistical analysis

Data was stored in Microsoft Excel and analysed using PRISM 10 (San Diego, California, USA). Demographic data, feedback forms and descriptive data will be reported using means and SD for continuous data. Absolute number and percentages will be reported for categorical data. Any statistical software can be used. For reference events (eg, apnoeas) or respiratory metrics (eg, RR), Root Mean Square Deviation to quantify the accuracy of manually annotated biosensor data (and developmental algorithm results) compared with standard care. A target accuracy of ±2 bpm/% of RR will be predefined as clinically acceptable error margin. Bland-Altman analysis will additionally be performed to visualise the bias between measurements. For respiratory event identification (eg, central apnoea), a confusion matrix stating the true positive (TP), false positive (FP) and false negative (FN) values of biosensor data (and developmental algorithm detection) of a specific respiratory event will be generated. Receiver operating characteristic curve analysis will be undertaken to evaluate the predictive performance of algorithms for evaluating central apnoeas in CR-P patients.

### Biosensor analysis

Biosensors data will be interpreted blinded to CR-P data scored by physiologists according to AASM guidelines to evaluate potential for apnoea detection and interrogation of RR and pattern using RIP bands as ground truth. TcCO_2_ data provide an indicator of hypoventilation.

For neonatal patients, biosensor data will be paired with oximetry data and viewed alongside SpO_2_ trace, recorded RR observation and ventilator measured parameters when available.

For patients with epilepsy, biosensor data will be interpreted in the context of VT, EEG evidence of epileptic seizures and video recording for breathing effort in the peri-ictal period.

### Algorithm under test

Developmental versions of central apnoea algorithm/actigraphy algorithm, RR algorithm.

### Data management

Site-specific data including consent forms, ethics documents and CRFs will be stored securely in the clinical research facility at the Queen Elizabeth University Hospital, Glasgow. An Excel spreadsheet which contains patient identifiable information (eg, Community Health Index number) and participant study number will be stored on an NHS computer, password protected and relevant only to individuals in the study team who are permitted to access clinical information.

Anonymised accelerometer data (using study number as reference) from PneumoWave DCM will be stored on a data storage platform (supplied by Galen Data) complete with Health Insurance Portability and Accountability Act requirements. Additionally, Galen Cloud deployed on Amazon Web Services is Health Information Trust Alliance certified.

### Study monitoring/auditing

An annual progress report will be submitted to the funder. Annual reports will be submitted to the ethics committee and sponsor with the first submitted 1 year after the date that all trial-related approvals are in place. This study will be risk assessed by NHS Greater Glasgow and Clyde Health Board Research and Development department, and the audit of this study will be determined by the results of the risk assessment.

### Early withdrawal

Participants may request to withdraw from the study at any stage for any reason without prejudice. Data up until the point of withdrawal will be kept. Should an individual choose to withdraw from the study, no further data will be collected.

### Study procedure risks and benefits

Anticipated risks with biosensor monitoring are expected to be minimal. This is a minimally invasive intervention with no protocol-specified procedures impacting the standard of care. Biosensor data will be analysed retrospectively in parallel with standard care data collected from CRF. Potential study risks include data breaches. However, all electronic data will be anonymous and stored on secure, password-protected devices or servers, and accessible only by approved study personnel. Numerical study IDs will be used in place of participant name.

There is a risk that wearing the sensor may cause distress, especially if patients are already in an unfamiliar environment, sensory processing disorders, concurrent stresses such as seizures from withdrawing antiepileptic medication during VT. Additionally, there may be a risk of skin irritation from the ECG sticker which could not have been foreseen, particularly for VT patients wearing the sensor for consecutive days. The ECG stickers used in this study are designed to be worn for 1 day continuously and will be changed daily, checking skin for irritation.

This study aims to support the development of a future device which can improve respiratory monitoring and apnoea detection for a diverse range of paediatric and neonatal patients in the future. The absence of immediate benefit for the participant will be outlined during the consent process.

### Safety

The capture of safety events will be solely related to adverse device effects (ADEs) and will only be captured for an event relating to the use or wearing of the device. All ADEs will be assessed for severity and reported to the sponsor team.

All ADEs and serious ADEs will be recorded from the time a participant is enrolled until the participant’s monitoring period ends. Participants who have enrolled but then withdrawn prior to wearing the biosensor for the first time will not have adverse events captured. Only unanticipated effects will be captured as reportable ADEs.

### Patient and public involvement

None.

## Ethics and dissemination

The study will be carried out in accordance with the World Medical Association Declaration of Helsinki (1964) standard reporting. Favourable ethical opinion was granted by the Regional Ethics Committee Leeds (IRAS 314696). Patients will only be allowed to enter the study once they have provided assent/consent where appropriate and parental consent has been obtained. Children aged 6–11 years attending for CR-P or VT were eligible to assent to their involvement in the study if able to understand, retain and weigh up information provided in the participant information sheet (online supplemental material 1). Children aged 12–16 years were eligible to consent if they were felt to be able to understand, retain and weigh up information to make an informed decision (online supplemental material 2). Written consent was required from a parent/guardian of each child (onlinesupplemental materials 3  4). Parents/guardians provided consent for neonatal patients. The Chief Investigator will be responsible for updating the Ethics committee of any new information related to the study. The study is registered at ClinicalTrials.gov. Study findings will be reported clearly and transparently with relevant stakeholders including researchers, practitioners and publicly available databases. Results from this study will be presented at national and international conferences and published in peer-reviewed journals.

## Discussion

This protocol will provide initial insights into the feasibility of collecting respiratory data from a wireless wearable biosensor in different patient groups where respiratory monitoring and apnoea detection is critical yet can be challenging. Data collected will be used to identify ‘biomarkers’ to accurately detect respiratory function. Evaluation of RR, pattern and apnoea detection is conceptually simple; however, in clinical practice, they can be challenging due to patient size, tolerance of standard monitoring equipment and reliance on manual interpretation by the clinical team.^37^

Wearable technology provides opportunity to reduce health inequalities, widening access to respiratory monitoring and diagnostic evaluation of SDB. The wireless nature of the biosensor data transmission overcomes geographical boundaries, such as in rural locations in Scotland and non-specialist centres without access to CR-P. Improving the acceptability of diagnostic equipment for patients with neurodevelopmental disorders who find tolerating standard equipment challenging is fundamental to achieving health equity.^1 38 39^

Wearable technology provides an opportunity to harness information from a simple metric (eg, RR and pattern). Application of machine learning may allow for identifications of early clinical deterioration, providing opportunity for intervention. Automated evaluation of respiratory events could reduce the burden of manual interpretation of CR-P and RR. Additionally, there is potential for a closed loop system where therapeutic intervention could be initiated following identification of a respiratory ‘biomarker’ or trend in respiratory pattern, such as patients requiring non-invasive ventilation to support their breathing.

This feasibility study has multiple strengths; we will evaluate the acceptability of this wearable sensor in a tertiary children’s hospital alongside standard clinical care. We have used a broad eligibility criteria and will include a diverse range of patients, including those with comorbidities and neurodevelopmental disorders, strengthening the external validity of this study. Patients included in this study are at risk of apnoeas, respiratory failure or sudden unexpected death, evaluating feasibility in a population who may benefit from continuous respiratory monitoring with a wearable biosensor. No restrictions were placed on normal activities such as feeding, cares and skin to skin for neonatal patients which may introduce movement artefact, enabling real-world testing. Feedback from patients and parents will be valuable and guide necessary modifications to optimise comfort and acceptability for patients and families.

Limitations exist in this study. First, movement artefact may limit visualisation of respiratory waveform at times; however, this is representative of real-world use and actigraphy as a metric can be useful for tracking clinical condition. Second, issues with data capture may occur; for example, ECG electrodes may be displaced. However, this is not directly related to the device under test (PneumoWave adhesive in the development pipeline).

Innovation within paediatric respiratory monitoring is required as existing devices are poorly tolerated by many children, resulting in high rates of incomplete and inconclusive investigations. Development of user-friendly and cost-effective devices offers additional opportunities to increase the duration and frequency of investigative periods. Wearable devices provide opportunity for longitudinal monitoring for the detection of life-threatening respiratory deterioration in real-time and alerting of carers to improve survivability. Future development of this work will involve conducting randomised clinical investigations of the biosensor to evaluate the potential integration of the biosensor within clinical pathways and collect health economics and outcomes data. Non-inferiority studies will determine the effectiveness of algorithms, for example, respiratory event identification in children attending for SDB evaluation. Larger scale studies monitoring patients with epilepsy at risk of SUDEP such as Dravet’s syndrome will provide additional information around respiratory changes in the peri-ictal period, currently poorly understood due to the difficulties of data capture with existing devices in this patient group. Bespoke neonatal algorithms can be tested and validated against standard clinical monitoring data.

## Supplementary material

10.1136/bmjopen-2025-104363 online supplemental file 1

10.1136/bmjopen-2025-104363 online supplemental file 2

10.1136/bmjopen-2025-104363 online supplemental file 3

10.1136/bmjopen-2025-104363 online supplemental file 4
