# Supplementary material for: Respiratory monitoring and apnoea detection in paediatric and neonatal patients using a wearable accelerometer-based chest sensor: protocol for an observational diagnostic feasibility study
Source: BMJ Open. 2025 Aug 31;15(8):e104363. doi: 10.1136/bmjopen-2025-104363 (PMC12406817; doi:10.1136/bmjopen-2025-104363)
Supplement: online supplemental file 1 [file bmjopen-15-8-s001.docx]

**“**The PARS Study: Paediatric Advanced Respiratory Service Study**”**

Researcher – Dr Ross Langley, NHS Greater Glasgow and Clyde

Please circle your answer:

Have you read or has somebody else explained this project to you? Yes / No

Do you understand what this project is about? Yes / No

Have you asked all the questions you want? Yes / No

Have you had your questions answered in a way that you understand? Yes / No

Do you understand that it’s OK to stop taking part at any time? Yes / No

Are you happy to take part? Yes / No

If **any** answers are ‘**no**’ or you don’t want to take part, please don’t sign your name.

If you **do** want to take part, please sign your name below

Child or parent name___________________________________

Signature____________________________________________

Date________________________________________________

The person who explained this project to you should sign here:

Print name___________________________________________

Signature____________________________________________

Date________________________________________________

① Participant copy; ② Site file copy; ③ Patients notes copy

If found please contact Ross Langley: email: ross.langley@ggc.scot.nhs.uk, tel.: 07736322142
