# Supplementary material for: Respiratory monitoring and apnoea detection in paediatric and neonatal patients using a wearable accelerometer-based chest sensor: protocol for an observational diagnostic feasibility study
Source: BMJ Open. 2025 Aug 31;15(8):e104363. doi: 10.1136/bmjopen-2025-104363 (PMC12406817; doi:10.1136/bmjopen-2025-104363)
Supplement: online supplemental file 2 [file bmjopen-15-8-s002.docx]

**CONSENT FORM: Main Study**

**Study Title:** The PARS Study: Paediatric Advanced Respiratory Service Study

Participant Name Participant Study Number

|  | Initial box |
| --- | --- |
| I confirm that I have read the patient information sheet dated 05/02/2024 (version 1.0) for the above study and confirm I have had the opportunity to consider the information, ask questions and have these answered satisfactorily. |  |
| I understand that my participation is voluntary and that I am free to withdraw at any time, without my medical care or legal rights being affected. |  |
| I understand that the NHS GG&C research team will have access to my medical records. |  |
| I understand that any information recorded in this study will remain confidential and that it may be looked at by representatives of the study Sponsor (NHS GGC) or regulatory bodies for audit purposes. |  |
| I understand that de-identified data will be used for research purposes, including machine-learning predictive modelling and device algorithm development analyses by commercial partners. |  |
| I understand that study data will be stored in NHS GG&C SafeHaven at the end of the study and may be used for future research purposes, with appropriate ethical approvals and your consent. |  |
| I understand that my anonymised data will be shared with colleagues at the University of Strathclyde for further analysis. |  |
| I understand that the anonymised data collected may be used in a research thesis, published in scientific literature and presented at scientific conferences. |  |
| I understand if I withdraw then previously captured data may still be utilised for the study purposes. |  |
| I agree to take part in the PARS study. |  |

Participant Date Signature

Name of person receiving consent Date Signature
