# Supplementary material for: Respiratory monitoring and apnoea detection in paediatric and neonatal patients using a wearable accelerometer-based chest sensor: protocol for an observational diagnostic feasibility study
Source: BMJ Open. 2025 Aug 31;15(8):e104363. doi: 10.1136/bmjopen-2025-104363 (PMC12406817; doi:10.1136/bmjopen-2025-104363)
Supplement: online supplemental file 3 [file bmjopen-15-8-s003.pdf]

CONSENT FORM: Parent/ Guardian

Study Title: The PARS Study: Paediatric Advanced Respiratory Service Study (Group 3)

________________________________ ____________

Participant Name Participant Study Number

	Initial box
I confirm that I have read the patient information sheet dated 28/10/2024 (version 1.2) for the above study and confirm that I have had the opportunity to consider the information, ask questions and have these answered satisfactorily.	
I understand that the participation of my child/person I am consenting for is voluntary and that I am free to withdraw their participation at any time, without their medical care or legal rights being affected.	
I understand that the NHS GG&C research team will have access to the medical records of my relative/person I am consenting for.	
I understand that any information recorded in this study will remain confidential and that it may be looked at by representatives of the study Sponsor (NHS GGC) or regulatory bodies for audit purposes.	
I understand that de-identified data of my child/person I am consenting for will be used for research purposes, including machine-learning predictive modelling and device algorithm development analyses by commercial partners.	
I understand that the anonymised data collected will be shared with colleagues at the University of Strathclyde for further analysis.	
I understand that study data will be stored in NHS GG&C SafeHaven at the end of the study and may be used for future research purposes, with appropriate ethical approval and consent.	
I understand that the anonymised data collected may be used in a research thesis, published in scientific literature and presented at scientific conferences.	
I understand if I withdraw from the study previously captured data may still be utilized for the study purposes.	
I agree to my child/person I am consenting for taking part in the PARS study.	

I can confirm that I am the Parent/Guardian for ____________________________and have the ability to provide consent on their behalf

Relationship to participant ________________________________

________________________________ ___________ ____________________________

Name of person giving consent Date Signature

________________________________ ___________ ____________________________

Name of person receiving consent Date Signature
