# Supplementary material for: Respiratory monitoring and apnoea detection in paediatric and neonatal patients using a wearable accelerometer-based chest sensor: protocol for an observational diagnostic feasibility study
Source: BMJ Open. 2025 Aug 31;15(8):e104363. doi: 10.1136/bmjopen-2025-104363 (PMC12406817; doi:10.1136/bmjopen-2025-104363)
Supplement: Supplementary file 5 [file bmjopen-2025-104363.reviewer_comments.pdf]

# PEER REVIEW HISTORY

BMJ Open publishes all reviews undertaken for accepted manuscripts. Reviewers are asked to complete a checklist review form and are provided with free text boxes to elaborate on their assessment. These free text comments are reproduced below.

## ARTICLE DETAILS

### Title (Provisional)

Respiratory monitoring and apnoea detection in paediatric and neonatal patients using a wearable accelerometer-based chest sensor: protocol for an observational diagnostic feasibility study

### Authors

Vennard, Hannah; Buchan, Elise; Miller, Jennifer; Kelly, Stuart; Cowan, Catriona; Meredith, Osian; Henderson, Bruce; Lowe, David J; Patel, Neil; Zuberi, Sameer; Langley, Ross

---

## VERSION 1 - REVIEW

---

| | |
|---|---|
| **Reviewer** | **1** |
| **Name** | **DALAI, RICHIE** |
| **Affiliation** | **All India Institute of Medical Sciences - Patna, Neonatology** |
| **Date** | **11-Jun-2025** |
| **COI** | **None** |

---

The type of study in the title should be very specific. Just writing observational study doesn't clarify the type of observational study.

Kindly clarify the "groups" mentioned in methods under the abstract section.

Why were the neonates less than 30 weeks gestation excluded?

Apneas are more seen in less than 30 weeks. Why not do a subgroup analysis instead?

The data of those on invasive mechanical ventilation or on NIPPV, are not comparable with those without any support or on CPAP. These should have subgroup analysis or the prior should be excluded.

Need to specify the statistical software that is planned to be used in a citable format.

Need to specify the statistical tests and the variables/ outcome measures to be analyzed in more detail.

Need to specify that the written consent will be obtained from LAR (legally authorized representative/ guardian) as these are children.

| | |
|---|---|
| **Reviewer** | **2** |
| **Name** | **Ibarra-Rios, Daniel** |
| **Affiliation** | **Hospital Infantil de Mexico Federico Gomez, Neonatology** |
| **Date** | **23-Jun-2025** |
| **COI** | **None** |

Thank you for letting me review this study protocol for a feasibility study of a chest worn accelerometer-based motion sensor.

In the introduction it would be important to point out controversies around home monitoring and SIDS as its currently not recommended [Sodini C, Paglialonga L, Antoniol G, Perrone S, Principi N, Esposito S. Home Cardiorespiratory Monitoring in Infants at Risk for Sudden Infant Death Syndrome (SIDS), Apparent Life-Threatening Event (ALTE) or Brief Resolved Unexplained Event (BRUE). Life (Basel). 2022 Jun 13;12(6):883. doi: 10.3390/life12060883]. In the newborn population it's important to specify more thoroughly inclusion criteria for newborns other than more than 30 week's gestation. As this study's objective is to truly reflect population of interest at risk, what would be reasons for exclusion as you state (clinical opinion of the investigator the patient is not suitable for inclusion) to capture the population of interest from the broad spectrum of neonates admitted in the neonatal unit.

In Figure 1 instead of a cartoon a clinical image would be more illustrative. For clarity I would always refer to two pediatric and one neonatal group as in study design where you state the 3 groups is confusing: CR-P (sleep study), neonates and VT (epilepsy). For clarity in the text and in Figure 2 I would separate: 1) children attending for cardiorespiratory sleep studies, 2) children with epilepsy attending for video telemetry and 3) newborns staying in the neonatal unit or post-natal ward.

Regarding sample size the number of patients was calculated but I wonder if time monitored needs to be considered. Especially as in the newborn population is stated that the sensor would be used in periods from 1-6 hours so is there any consideration about the monitoring time needed to draw conclusions.

Is there a specific score to assess skin tolerability in newborn like the Neonatal Skin Condition Score?

Again, in discussion I would mention controversies as monitoring hasn't proved to decrease SIDS and catastrophic outcomes from unexpected collapse pointing out the strengths of this study approach.

---

## VERSION 1 - AUTHOR RESPONSE

Reviewer: 1
Dr. RICHIE DALAI, All India Institute of Medical Sciences - Patna
Comments to the Author:
The type of study in the title should be very specific. Just writing observational study doesn't clarify the type of observational study.
RESPONSE: Amended, Observational diagnostic feasibility study

Kindly clarify the "groups" mentioned in methods under the abstract section.
RESPONSE: Clarified, 'We aim to recruit 3 groups of patients and 75 patients to each group: (1) Children attending for overnight cardiorespiratory polygraphy; (2) neonatal patients, (3) children attending for video telemetry at the epilepsy monitoring unit. '

Why were the neonates less than 30 weeks gestation excluded?
Apneas are more seen in less than 30 weeks. Why not do a subgroup analysis instead?
RESPONSE: We acknowledge that neonates < 30 weeks gestation at greater risk of apnoeas. As this is a feasibility study we decided 30 weeks corrected gestation and older would be more appropriate given this sensor was initially designed for adults the size may need to be reduced for neonates < 30 weeks. At this stage we are focusing on tolerability and feasibility. We aim to test limitations of the device in its current form any future studies would test feasibility in the subgroup you have correctly identified as having an increased risk of apnoea. Furthermore, the target population for neonates are likely babies who are on the post-natal ward largely unmonitored. Neonates having sleep studies are being recruited into this study who are at increased risk of apnoeas.

The data of those on invasive mechanical ventilation or on NIPPV, are not comparable with those without any support or on CPAP. These should have subgroup analysis or the prior should be excluded.
RESPONSE: Data from patients receiving respiratory support will be analysed separately

Need to specify the statistical software that is planned to be used in a citable format.
Need to specify the statistical tests and the variables/ outcome measures to be analysed in more detail.
RESPONSE: Added details of statistical software used 'Data was stored in Microsoft excel and analysed using PRISM 10 (San Diego, California, USA)'. Receiver operating characteristic (ROC) curve analysis will be undertaken to evaluate the predictive performance of algorithms for evaluating central apnoeas in cardiorespiratory polygraphy patients.

Need to specify that the written consent will be obtained from LAR (legally authorized

representative/ guardian) as these are children.
RESPONSE: Amended to provide further details of assent/ consent as outlined above.

Reviewer: 2
Dr. Daniel Ibarra-Rios, Hospital Infantil de Mexico Federico Gomez
Comments to the Author:
Thank you for letting me review this study protocol for a feasibility study of a chest worn accelerometer-based motion sensor.

In the introduction it would be important to point out controversies around home monitoring and SIDS as its currently not recommended [Sodini C, Paglialonga L, Antoniol G, Perrone S, Principi N, Esposito S. Home Cardiorespiratory Monitoring in Infants at Risk for Sudden Infant Death Syndrome (SIDS), Apparent Life-Threatening Event (ALTE) or Brief Resolved Unexplained Event (BRUE). Life (Basel). 2022 Jun 13;12(6):883. doi: 10.3390/life12060883].
RESPONSE: Added, Controversies exist around home monitoring for Sudden unexpected death in infants however cardiorespiratory monitoring following a previous brief resolved unexplained event can be considered.(28)

In the newborn population it's important to specify more thoroughly inclusion criteria for newborns other than more than 30 week's gestation. As this study's objective is to truly reflect population of interest at risk, what would be reasons for exclusion as you state (clinical opinion of the investigator the patient is not suitable for inclusion) to capture the population of interest from the broad spectrum of neonates admitted in the neonatal unit.
RESPONSE: As above, we acknowledge that neonates < 30 weeks gestation at greater risk of apnoeas. As this is a feasibility study we decided 30 weeks corrected gestation and older would be more appropriate given this sensor was initially designed for adults the size may need to be reduced for neonates < 30 weeks. At this stage we are focusing on tolerability and feasibility. We aim to test limitations of the device in its current form any future studies would test feasibility in the subgroup you have correctly identified as having an increased risk of apnoea. Furthermore, the target population for neonates are likely babies who are on the post-natal ward largely unmonitored. Neonates having sleep studies are being recruited into this study who are at increased risk of apnoeas.

In Figure 1 instead of a cartoon a clinical image would be more illustrative.
RESPONSE: We feel a stylised image is clearer to demonstrate data workflow. Additionally, there are issues around photographic consent in children.

For clarity I would always refer to two paediatric and one neonatal group as in study design where you state the 3 groups is confusing: CR-P (sleep study), neonates and VT (epilepsy).
RESPONSE: Children in the CR-P and VT group can be 0-16 years and therefore may be in the neonatal period therefore we feel it is clearer to have three distinct groups instead of two paediatric and one neonatal. Children attending for cardiorespiratory polygraphy (CR-P) and children attending for video telemetry will have different standard monitoring equipment (ground truth for novel biosensor interpretation) in addition to different respiratory events/ patterns. For example, children attending for (CR-P) are at increased risk of sleep disordered breathing with central and obstructive apnoeas, hypoventilation and will wear two biosensors

(chest and abdominal) to evaluate paradoxical breathing. Children attending for VT on the other hand will wear a single sensor.

For clarity in the text and in Figure 2 I would separate: 1) children attending for cardiorespiratory sleep studies, 2) children with epilepsy attending for video telemetry and 3) newborns staying in the neonatal unit or post-natal ward.
RESPONSE: Further clarity given to groups in Figure 2 legend, Group 1 include children attending for cardiorespiratory polygraphy; group 2 include neonates staying in the neonatal unit or postnatal ward; group 3 include children attending the epilepsy monitoring unit for video telemetry.

Regarding sample size the number of patients was calculated but I wonder if time monitored needs to be considered. Especially as in the newborn population is stated that the sensor would be used in periods from 1-6 hours so is there any consideration about the monitoring time needed to draw conclusions.
RESPONSE: As this is a feasibility study it was felt appropriate for participants to wear the sensor for a duration of time that would acceptable for patients and their parents/ guardian. Many neonates on the post-natal ward are only staying for a short period prior to discharge and require input from multiple members of the healthcare team including newborn examinations on this occasion a shorter duration of monitoring was felt appropriate. For neonates having oximetry studies the sensor was worn for longer durations (e.g. 6 hours). We feel 1-6 hour duration will provide ample data for determining the potential feasibility of this sensor across a diverse range of patients.

Is there a specific score to assess skin tolerability in newborn like the Neonatal Skin Condition Score?
RESPONSE: At present an ECG electrode is being used for attaching sensor to the patient's chest which is licensed for use in the paediatric population. Custom-made adhesive are in the development pipeline.

Again, in discussion I would mention controversies as monitoring hasn't proved to decrease SIDS and catastrophic outcomes from unexpected collapse pointing out the strengths of this study approach.
RESPONSE: Patients included in this study are at risk of respiratory failure or sudden unexpected death which further strengthens the external validity of this this observational diagnostic feasibility study evaluating its target population.

---

**VERSION 2 - REVIEW**

---

| | |
|---|---|
| **Reviewer** | **1** |
| **Name** | **DALAI, RICHIE** |
| **Affiliation** | **All India Institute of Medical Sciences - Patna, Neonatology** |
| **Date** | **14-Jul-2025** |
| **COI** | |

---

The authors have brought important changes to the manuscript. There are few other concerns that need to be addressed. These are as follows:

1) In the title page the superscripts for the authors and affiliations should match. "d" and "e" are written as "D" and "E" in the respective affiliations.

2) The exact population age group that is planned to be studied need to be mentioned in the abstract. Just writing children is not enough. Similarly the gestation age range of neonates being included needs to be mentioned in the abstract.

3) The strengths and limitations refer to strengths and limitations of the current study protocol compared to previous studies if available. This is not regarding the device. The three points written in strengths seem similar.

4) Putting a 20 grams sensor on the chest of a neonate may not be advisable as it itself may cause obstruction to the breathing efforts of the baby. How will these adverse events be recorded? How will they be differentiated from true apneas?

5) The authors need to elaborate on the potential adverse events including local skin reactions due to usage of ECG lead adhesive. How will the skin changes or dermatitis be scored depending on severity? And what will be the criteria for post enrollment exclusion apart from participant request?

---

## VERSION 2 - AUTHOR RESPONSE

Reviewer: 1
Dr. RICHIE DALAI, All India Institute of Medical Sciences - Patna
Comments to the Author:
The authors have brought important changes to the manuscript. There are few other concerns that need to be addressed. These are as follows:

1) In the title page the superscripts for the authors and affiliations should match. "d" and "e" are written as "D" and "E" in the respective affiliations.
RESPONSE: updated to lower case to match

2) The exact population age group that is planned to be studied need to be mentioned in the abstract. Just writing children is not enough. Similarly the gestation age range of neonates being included needs to be mentioned in the abstract.
RESPONSE: updated abstract to include age groups and gestational age of neonates being included

3) The strengths and limitations refer to strengths and limitations of the current study protocol compared to previous studies if available. This is not regarding the device. The three points written in strengths seem similar.
RESPONSE: we feel strengths are distinct, modified to emphasise the distinct nature of points (1) this study is taking place at a large tertiary children's hospital; (2) standard clinical monitoring data is being collected and provides a comprehensive ground truth for novel biosensor data interpretation; (3) patients recruited into this study are diverse in terms of comorbidities with broad eligibility criteria strengthening external validity and are also representententative of patients who may benefit from monitoring with a wearable sensor.

4) Putting a 20 grams sensor on the chest of a neonate may not be advisable as it itself may cause obstruction to the breathing efforts of the baby. How will these adverse events be recorded? How will

they be differentiated from true apneas?
Neonates wearing the sensor will be monitored by nursing staff and with standard clinical monitoring (oximetry and clinical observation). We do not anticipate any obstruction to be precipitated by the biosensor; the neonate will not be recruited unless the clinical team looking after the patient feels it is appropriate.

5) The authors need to elaborate on the potential adverse events including local skin reactions due to usage of ECG lead adhesive. How will the skin changes or dermatitis be scored depending on severity? And what will be the criteria for post enrollment exclusion apart from participant request?
RESPONSE: ECG adhesive used in this study is licenced for use in this patient population (used in standard clinical practice). Guidelines for use of ECG adhesives are being followed as per standard clinical care (e.g. duration of wear).
